# Methane-Rich Saline Alleviates CA/CPR Brain Injury by Inhibiting Oxidative Stress, Microglial Activation-Induced Inflammatory Responses, and ER Stress-Mediated Apoptosis

**DOI:** 10.1155/2020/8829328

**Published:** 2020-10-14

**Authors:** Ruixia Cui, Sinan Liu, Cong Wang, Tong Liu, Jie Ren, Yifan Jia, Yingmu Tong, Chang Liu, Jingyao Zhang

**Affiliations:** ^1^Department of Hepatobiliary Surgery, The First Affiliated Hospital of Xi'an Jiaotong University, Xi'an, Shaanxi 710061, China; ^2^Department of SICU, The First Affiliated Hospital of Xi'an Jiaotong University, Xi'an, Shaanxi 710061, China

## Abstract

Brain injury induced by cardiac arrest/cardiopulmonary resuscitation (CA/CPR) is the leading cause of death among patients who have recovery of spontaneous circulation (ROSC). Inflammatory response, apoptosis, and oxidative stress are proven pathological mechanisms implicated in neuronal damage. Methane-rich saline (MRS) has been proven that exerts a beneficial protectiveness impact in several models of ischemia-reperfusion injury. The goal of this paper is to ascertain the role of MRS in CA/CPR-induced brain injury and its potential mechanisms. The tracheal intubation of Sprague-Dawley (SD) rats was clamped for 6 min to establish an asphyxiating cardiac arrest model. After that, chest compressions were applied; then, MRS or saline was administered immediately post-ROSC, the rats were sacrificed, and brain tissue was collected at the end of 6 hours. We observed that MRS treatment attenuated neuronal damage in the hippocampal CA1 region by inhibiting microglial activation, leading to a decrease in the overexpression of proinflammatory cytokines such as TNF-*α*, IL-6, and iNOS. The results also illustrated that MRS treatment diminished apoptosis in the hippocampal CA1 region , reduced the expression of apoptosis-associated proteins Bax and cleaved caspase9, and increased Bcl-2 expression, as well as inhibited the expression of endoplasmic reticulum (ER) stress pathway-related proteins GRP78, ATF4, and CHOP. Further findings showed that MRS treatment significantly attenuated hippocampal ROS and MDA levels and increased GSH and SOD antioxidant factor levels, which indicated that MRS treatment could inhibit oxidative stress. Our results suggest that MRS exerts a protective effect against CA/CPR brain injury, by inhibiting oxidative stress, microglial activation-induced inflammatory responses, and ER stress-mediated apoptosis.

## 1. Introduction

Cardiac arrest (CA) is a significant clinical problem that challenges global public health. According to recent survey statistics, cardiac arrest affects upwards of 550,000 adults in the United States annually, contributing to more than a million deaths [1, 2]. Worse, it is associated with an in-hospital mortality rate of 65-90%, and only 5-17% of the patients with recovery of autonomous circulation (ROSC) after cardiopulmonary resuscitation (CPR) had not suffered serious neurological problems at discharge [3, 4]. The brain is considered the most sensitive organ to systemic ischemia-reperfusion and inflammatory response injury after CA/CPR [5]. Brain injury after CPR is the predominant cause of eventual death among ROSC patients [6].

The pathogenesis of brain injury after CA/CPR is complex and is currently accepted to be mainly related to inflammatory response, free radical formation, damage and death of cells, etc. [7–9]. Microglia are macrophage-like cells that reside in the brain and have been shown to play an essential role in the cerebral inflammatory response [10]. Increasing evidence shows that microglial activation in reperfusion injury induces the expression of a significant number of proinflammatory cytokines, for instance, interleukin-1*β* (IL-1*β*), tumor necrosis factor-*α* (TNF-*α*), and inducible nitric oxide synthase (iNOS) [8, 10]. Studies have shown that large amounts of oxygen free radicals (ROS) were produced after CA/CPR cerebral ischemia-reperfusion [11]. Cellular ROS overload leads to increased abnormal protein folding and triggers endoplasmic reticulum (ER) stress [12]. Then, glucose-regulating protein 78 (GRP78) is separated from the ER transmembrane receptor proteins PKR-like ER kinase (PERK), inositol-requiring enzyme 1 (IRE1*α*), and activating transcription factor 6 (ATF-6), which enhances ER degradation and triggers unfolded protein response (UPR), thereby enhancing cell viability. When the compensation of ER is saturated, the C/EBP homologous protein (CHOP) pathway is activated, upregulating apoptosis regulatory proteins and inducing cell apoptosis [13, 14]. Thus, it is possible that suppression of the activated microglial-induced inflammatory response and ER stress-mediated apoptosis may pose a promising therapeutic target for brain protection after CA/CPR.

An emerging star gas, methane, as the richest organic gas in nature, is colorless, odorless, and nontoxic and possesses reducibility, which has attracted increasing attention in recent years, especially for the treatment of diseases [15]. Studies indicate that methane plays a unique biological role in anti-inflammatory, antioxidation, antiapoptosis, and other cell protection, which has been confirmed in many different animal experiments [16–18]. Methane has been shown to exhibit protective effects in hepatic, myocardial, and renal ischemia-reperfusion injury through antioxidative, anti-inflammatory, and antiapoptotic effects [19–21]. A recent study of MRS on acute kidney injury caused by sepsis showed that MRS could restrain the CHOP signaling pathway regulated by endoplasmic reticulum stress, providing a protective effect [21]. However, no studies have been conducted on the effects of MRS in brain injury after CA/CPR. Therefore, this study intends to investigate the effectiveness of MRS against brain injury after CA/CPR and its underlying mechanisms, in order to develop alternative treatment approaches toward the early brain injury after CA.

## 2. Materials and Methods

### 2.1. Experimental Animals

Male Sprague-Dawley rats (weighing 280–300 g) were purchased from the Experimental Animal Center, Health Sciences Center, Xi'an Jiaotong University. Animals were kept in the clean animal room (ambient temperature 20-25°C, relative humidity 50%, and 12 h/12 h bright/dark cycle) and given regular diet and water. This study was authorized and conducted by the Ethics Committee Institution, the Health Sciences Center, Xi'an Jiaotong University, and followed the National Institutes of Health Guidelines for the Regulation of Laboratory Animal.

### 2.2. Methane-Rich Saline Preparation

Referring to the preparation method of Ye et al. [19], methane was prepared one day before the experiment by solubilizing it in sterile saline under high pressure (0.6 MPa) for 4 hours then stored at 4°C. The MRS was irradiated with *γ*-rays before use for disinfection. The concentrations of MRS in the range of 1.2-1.5 mmol/L were determined by gas chromatography (Gas Chromatography-9860, Qiyang, Shanghai, China).

### 2.3. Surgical Procedures and CA Model

**Figure 1:**
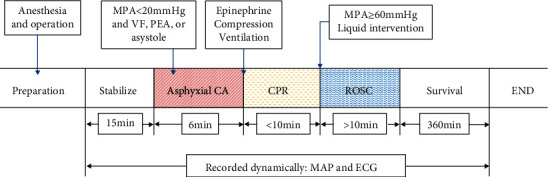
Experiment procedure flowchart. CA: cardiac arrest; CPR: cardiopulmonary resuscitation; ROSC: recovery of spontaneous circulation.

Rats were anesthetized by the administration of pentobarbital sodium (50 mg/kg) intraperitoneally. Anesthetized animals were fixed in a supine position on the surgical operating table. Hair was cleared from the neck and right groin area. After disinfection, the proximal trachea was intubated via a tracheostomy with a 14G cannula and mechanically ventilated by a tidal volume of 0.65 mL/100 g body weight and a respiratory rate of 100 breaths per minutes, absorption ratio (I : E) of 1 : 2, and fraction of inspired oxygen (FiO_2_) of 0.21 (ventilator: Tawang PV800, Shanghai, China). Continuously recorded standard II-lead electrocardiograms (ECG) were obtained by attaching acupuncture needles implanted under the skin of the limbs. The right femoral artery and vein were intubated with the PE-50 catheter, which was prefilled with 2.5 IU/mL of heparin sodium saline, to monitor blood pressure, liquid intervention, and drug administration. All of the physiological data were captured by the experimental protocol BL-420F Biological Signal Acquisition & Analysis System (Chengdu Taimeng Co., Ltd.). After 10-minute stabilization, animals were initiated cardiac arrest by clamping endotracheal intubation for 6 min. CA was defined as mean arterial pressure (MAP) < 20 mmHg, ventricular fibrillation (VF), pulseless electrical activity (PEA), or asystole, which typically appeared within 4 min. After 6 min of cardiac arrest, CPR was started by reconnecting mechanical ventilation (FiO_2_ = 1.0, other ventilator parameters were the same as before), performing chest compressions (180-200 beats/min), and administrating 0.02 mg/kg epinephrine. ROSC was defined as an organized rhythm recovery with MAP not less than 60 mmHg for a minimum of 10 min. Rats unable to ROSC within 10 minutes were excluded. The procedure of the experiment is shown in Figure 1.

### 2.4. Study Protocol

The rats were assigned to the four groups as follows (*n* = 12 per group): (1) sham control group, (2) MRS control group, (3) CPR+normal saline (NS) group, and (4) CPR+MRS group. Control group rats underwent all procedures except asphyxia CA/CPR. Concomitant to the start of ROSC, a bolus of 10 mL/kg of MRS or an equal volume of 0.9% NaCl was infused with a precision infusion set (Lange LSP01-2A, Hebei, China) via femoral vein for 2 hours; then, all catheters were removed. Mechanical ventilation was continued with 21% oxygen until the end of experiment after six hours. Finally, six rats in each group were immediately sacrificed; the hippocampus was rapidly dissected on ice and frozen at -80°C; the left hippocampus was applied for the detection of tissue factor and the right for western blotting. Another six rats were perfused through the heart with phosphate-buffered saline and 4% paraformaldehyde to obtain brain tissue.

### 2.5. Histologic Examination

The brain tissue that was perfused with paraformaldehyde was fixed in 10% formalin for 72 hours and then processed for embedding in paraffin. Consecutive 4 *μ*m thick slices were acquired from the paraffin and stained with Nissl staining solution. Then, two researchers assessed the injury degree of the hippocampal CA1 area in a double-blinded way through the light microscope (Olympus, Japan). Six fields were randomly selected, and viable neurons were counted. Viable neurons had complete cell morphology, with a distinct nucleus and nucleolus, and the Nissl corpuscles in the cytoplasm. Nonviable neurons exhibited shrunken in cell bodies, are irregular in shape, and showed nuclear pyknosis, karyorrhexis, and vacuolization.

### 2.6. Hippocampal Cytokine Levels and Oxidation Index Detection

The hippocampal tissue sample homogenate was prepared by centrifugation at 14,000g for 10 min using a phosphate buffer at 4°C. The supernatants of the homogenate were extracted and assayed by ELISA kits (Jiancheng Institute of Biotechnology, Nanjing, China). The level of GSH, SOD, and MDA and the concentrations of TNF-*α*, IL-6, and IL-10 were measured following the manufacturer's instructions at room temperature.

### 2.7. Immunohistochemical Analysis

The brain tissue paraffin sections were deparaffinized, rehydrated, and treated with citrate buffer for antigen retrieval. After that, the sections were sequentially incubated with 3% hydrogen peroxide, goat serum for 15 minutes, followed by the primary antibodies against NeuN (1 : 200, NOVUS, USA), TMEM119 (1 : 250, Proteintech, China), CD68 (1 : 200, Immunoway, China), and iNOS (1 : 500, Abcam, USA) incubation overnight at 4°C. The following day, after PBS washout, using biotinylated secondary antibodies incubated for 1 h, PBS was washed out again. Afterwards, diaminobenzidine tetrahydrochloride (DAB) was incubated to observe visualization by microscopy.

### 2.8. Immunofluorescence Staining

The brain tissue cryosections were routinely thawed and washed. Then, the primary antibody Iba1 (1 : 500, Abcam, USA) was incubated overnight at 4°. After the second day, PBS washed, Alexa Fluor 488-conjugated goat anti-rabbit secondary antibodies were incubated for 1 h (1 : 300, Servicebio, China). Subsequently, cell nuclei were counterstained with 4′-6-diamino-2-phenylindole (DAPI) followed by observation and photography. Fluorescence images were acquired using the Inverted Fluorescence Stereomicroscope (Axio Observer A1, Zeiss, Germany). The blue and green signals were observed under purple (405 nm) and blue (488 nm) excitation light, respectively.

### 2.9. Apoptosis Assay

The brain tissue cryosections were examined with a fluorescence detection kit (In Situ Cell Death Detection Kit; Fluorescein, Roche, Switzerland) for TUNEL staining according to product instructions. The green spots excited at 488 nm indicated positive apoptotic cells as observed by the Inverted Fluorescence Stereomicroscope (Axio Observer A1, Zeiss, Germany).

### 2.10. Detection of ROS Activation

As previously described, the brain tissue cryosections were placed at room temperature for 10 min, washed with PBS, and incubated with dihydroethidium (DHE, 10 *μ*M) (Vigorous Biotechnology Co. Ltd., Beijing, China) for 60 min at 37°C in the dark [21]. ROS was positive in the cells displayed as red fluorescence under green wavelength (594 nm) excitation under the Inverted Fluorescence Stereomicroscope (Axio Observer A1, Zeiss, Germany).

### 2.11. Western Blot Assay

The protein was extracted from fresh-frozen hippocampal tissue using RIPA lysate. The BCA method was used to measure protein concentration. The protein solution and SDS-PAGE loading buffer (5x) were mixed at 1 : 4, then heated at 100°C for 10 min to denature the protein. After this production, equal amounts of proteins were separated using SDS-PAGE gel and transferred onto the PVDF membrane. Then, PVDF membranes were blocked with 10% skim milk and incubated with anti-GRP78 antibody (1 : 5000, Proteintech, China), anti-ATF4 antibody (1 : 2000, Proteintech, China), anti-Bax antibody (1: 5000, Proteintech, China), anti-Bcl2 antibody (1 : 2000, Immunoway, China), anti-CHOP antibody (1 : 1000, Proteintech, China), caspase9 (1 : 1000, CST, USA), TNF-*α* (1 : 1000, Proteintech, China), IL-6 (1 : 1000, Proteintech, China), IL-10 (1 : 1000, Proteintech, China), and anti-*β*-actin antibody (1 : 10000, Santa Cruz, USA) overnight at 4°C. Afterwards, the bolts were incubated for 1 hour at 37°C using the corresponding anti-rabbit and anti-mouse HRP-conjugated secondary antibodies (1 : 10000, Proteintech, China). Finally, the protein bands were identified using the ECL Chemiluminescence Kits (Millipore, USA). Protein levels were normalized to *β*-actin as reference. The relative density of the protein level was quantitated by the ImageJ software.

### 2.12. Statistical Analysis

The data was presented as mean values ± standard deviation (SD). Differences among multiple groups were compared using one-way ANOVA with Dunn's multiple comparison test. Statistical significance was considered to be *p* < 0.05. Statistical analysis and graph layout were achieved with GraphPad Prism8.4 (GraphPad Software, Inc., LaJolla, CA, USA).

## 3. Results

### 3.1. Baseline Characteristics

**Figure 2:**
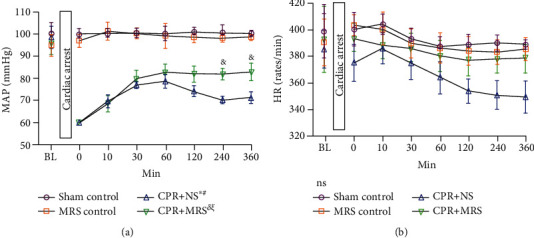
Changes in mean arterial pressure and heart rate during ROSC after CA: (a) mean arterial pressure (MAP) and (b) heart rate (HR). Bars represent the mean and SEM. BL indicates baseline. The statistical significance was assessed by the one-way ANOVA test (^*δ*^*p* < 0.05, and ^∗^*p* < 0.01 vs. sham control group; ^*ξ*^*p* < 0.05 and ^#^*p* < 0.01 vs. MRS control group; ^&^*p* < 0.05 and ^$^*p* < 0.01 vs. CPR+NS group; ns: no statistically significant difference).

Baseline characteristics and CA/CPR relevant parameters are shown in Table 1. There were no notable differences among the groups in baseline weight, heart rate, and MAP. In addition, no significant statistical difference was found in the asphyxia time, the successful resuscitation time, the success rate of resuscitation, and the mortality rate after resuscitation between the CPR+NS group and the CPR+MRS group. The MAP in the CPR group was obviously lower than that in the control group, while the MAP of the CPR+MRS group was significantly higher than that of the CPR+NS group at 240 and 360 min after ROSC (*p* < 0.05). Among the groups, no statistically significant differences were found in heart rate at any corresponding time point (Figure 2). However, we observed that MRS treatment allowed the blood pressure and heart rate of ROSC rats to reach a stable state earlier.

### 3.2. MRS Rescued Neuronal Damage in the Hippocampus after CA/CPR

**Figure 3:**
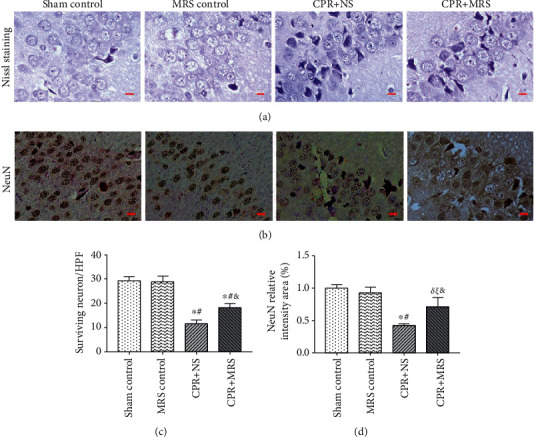
MRS rescued hippocampal neuronal damage after CA/CPR. (a) Representative photomicrographs of Nissl staining showing neurons. (b) Immunohistochemical staining of antineuronal nucleus antibody NeuN to detect neuronal survival status. (c) Surviving neurons counting per high-power field (×400). (d) Quantification of the NeuN-positive relative intensity area (%). Scale bars indicated 25 *μ*m. Data were reported as mean ± SD. The statistical significance was assessed by the one-way ANOVA test (^*δ*^*p* < 0.05, and ^∗^*p* < 0.01 vs. sham control group; ^*ξ*^*p* < 0.05 and ^#^*p* < 0.01 vs. MRS control group; ^&^*p* < 0.05 and ^$^*p* < 0.01 vs. CPR+NS group).

Studies have shown that the hippocampal CA1 region was a fragile region sensitive to cerebral ischemia and hypoxia, associated with neuronal damage and microglial activation in the early stage [22]. Therefore, we firstly evaluated neuronal damage by performed Nissl staining and NeuN immunohistochemical staining. The results of Nissl staining showed that the surviving neurons in the control group were well arranged and dense, with a visible nucleus, intact cytoplasm, and discernable outline. In the CPR challenge group, the number of cell layers was reduced and disordered, and a large number of deformed pyramidal cells with deeply stained and dense nuclei were visible in the hippocampal CA1 region. Compared with the CPR challenge group, the number of normal neurons in the MRS treatment group has increased and the arrangement is more regular, obviously preventing the injury of the CA1 neuron (*p* < 0.05) (Figures 3(a) and 3(c)). The immunohistochemical results showed that the number of surviving NeuN-positive cells was significantly lower than the control group; MRS treatment partly rescued the loss of NeuN-positive cells in CA/CPR rats (*p* < 0.05) (Figures 3(b) and 3(d)).

### 3.3. MRS Inhibited Microglial Activation in the Hippocampus after CA/CPR

**Figure 4:**
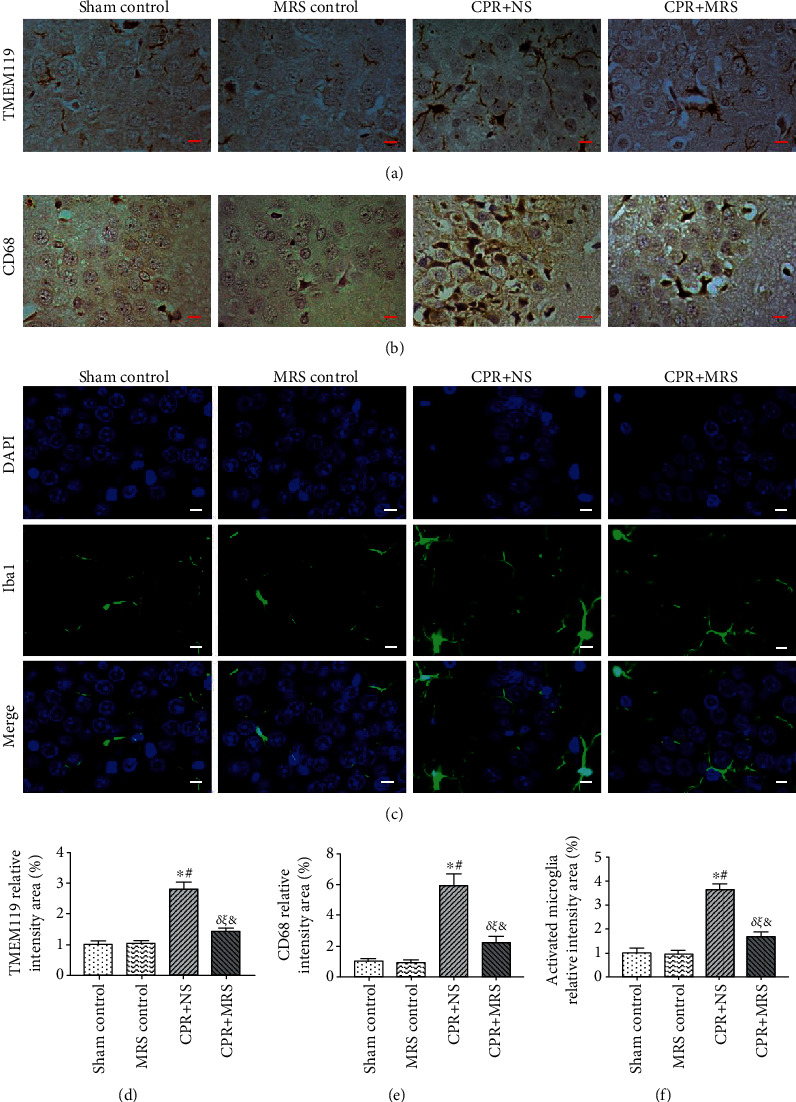
MRS inhibited microglial activation in the hippocampus after CA/CPR: (a) TMEM119 immunohistochemical staining, (b) CD68 immunohistochemical staining, and (c) Iba1 immunofluorescence to assess microglial activation status; (d) quantification of TMEM119 relative intensity area (%); (e) quantification of CD68 relative intensity area (%); (f) activated microglial relative intensity area (%). Data were reported as the mean ± SD. The statistical significance was assessed by the one-way ANOVA test (^*δ*^*p* < 0.05, and ^∗^*p* < 0.01 vs. sham control group;^*ξ*^*p* < 0.05 and ^#^*p* < 0.01 vs. MRS control group; ^&^*p* < 0.05 and ^$^*p* < 0.01 vs. CPR+NS group).

We intend to further clarify the state of microglial activation among the different groups. Resting microglia have smaller, highly branched cytosol, while activated microglia appeared to have fewer branches, shorter and thicker projections, enlarged cytosol, and rounded or rod-shaped cell morphology [23]. The immunohistochemical results revealed that compared with the control groups, more TMEM119-positive activated microglial cells and CD68-positive activated microglial cells were observed in the hippocampal CA1 region after CA/CPR (*p* < 0.01), while MRS treatment significantly inhibited microglial activation (*p* < 0.05) (Figures 4(a), 4(b), 4(d), and 4(e)). The Iba1-immunofluorescence results also indicated that MRS treatment suppressed microglial activation after CPR challenge (*p* < 0.01) (Figures 4(c) and 4(f)).

### 3.4. MRS Alleviated the Inflammatory Response in the Hippocampus after CA/CPR

**Figure 5:**
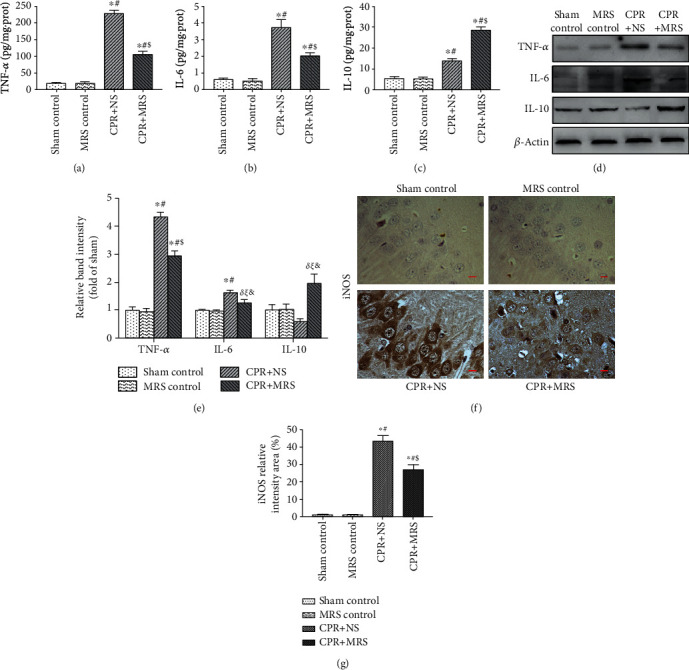
MRS alleviated the inflammatory response in the hippocampus after CA/CPR. The levels of (a) TNF-*α*, (b) IL-6, and (c) IL-10 in hippocampal tissues were measured 6 h after CA/CPR using commercial ELISA kits. (d) The expression levels of TNF-*α*, IL-6, and IL-10 in hippocampal tissue protein at 6 h after CA/CPR. (e) Relative band intensity of TNF-*α*, IL-6, and IL-10. (f) iNOS immunohistochemistry staining to detect hippocampal CA1 region inflammation response. (g) Quantification of iNOS relative intensity area (%). Scale bars indicated 25 *μ*m. Data were reported as mean ± SD. The statistical significance was assessed by the one-way ANOVA test (^*δ*^*p* < 0.05, and ^∗^*p* < 0.01 vs. sham control group; ^*ξ*^*p* < 0.05 and ^#^*p* < 0.01 vs. MRS control group; ^&^*p* < 0.05 and ^$^*p* < 0.01 vs. CPR+NS group).

The inflammatory response plays a significant role in brain injury, and studies have shown that activated microglia promote the release of inflammatory factors [24, 25]. Furthermore, previous studies suggested that MRS exerted an anti-inflammatory effect in organism damage [21]. Therefore, we further detected the expression of inflammatory factors in the hippocampal tissues of rats. The levels of TNF-*α*, IL-6, and IL-10 were assessed by ELISA (Figures 5(a)–5(c)) and were validated by western blot (Figures 5(d) and 5(e)). Results showed that the levels of TNF-*α* and IL-6 were markedly increased post-CA/CPR, while MRS treatment significantly downregulated the TNF-*α* and IL-6 inflammatory factor level and upregulated the IL-10 anti-inflammatory level (*p* < 0.05). Immunohistochemical analysis of the inflammatory marker iNOS yielded similar results to WB and ELISA results (Figures 5(f) and 5(g)). Compared with control groups, iNOS-positive cells obviously increased after CPR challenge, while MRS treatment significantly reduced iNOS expression (*p* < 0.01). According to those results, we found that MRS could reduce the inflammation response caused by CA/CPR.

### 3.5. MRS Reduced the Apoptosis Effect in the Hippocampus after CA/CPR

**Figure 6:**
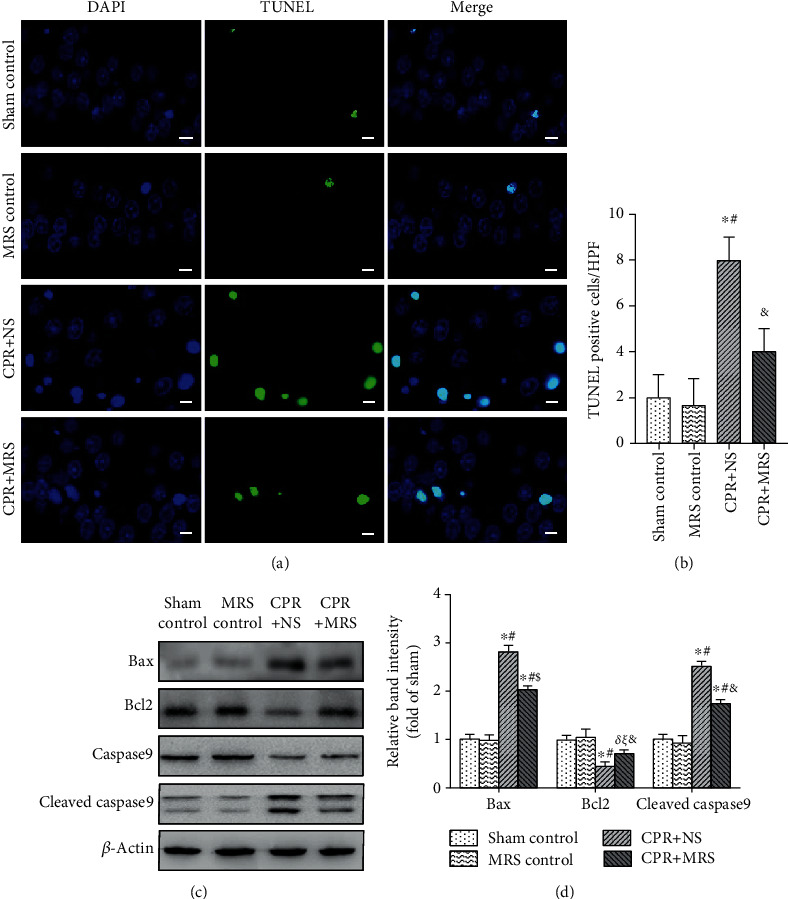
MRS reduced the hippocampal neuron apoptosis after CA/CPR. (a) TUNEL assay. (b) TUNEL-positive cells per high-power field (×400). (c) The expression levels of Bax, Bcl_2_, caspase9, and cleaved caspase9 in the hippocampus. (d) The relative band intensities (fold of the sham control group). Scale bars indicated 25 *μ*m. Data were reported as mean ± SD. The statistical significance was assessed by the one-way ANOVA test (^*δ*^*p* < 0.05, and ^∗^*p* < 0.01 vs. sham control group; ^*ξ*^*p* < 0.05, and ^#^*p* < 0.01 vs. MRS control group; ^&^*p* < 0.05 and ^$^*p* < 0.01 vs. CPR+NS group).

To clarify the effect of MRS on apoptosis in cerebral ischemia-reperfusion damage, we performed TUNEL staining. It revealed that TUNEL-positive cells were significantly increased in the CPR group, whereas MRS treatment reversed this change (*p* < 0.05) (Figures 6(a) and 6(b)). We further used western blot analysis of apoptosis-related proteins, including Bax, Bcl2, and cleaved caspase9. Consistent with the results of TUNEL, the CPR challenge group significantly enhanced the expression of the proapoptotic protein Bax and cleaved caspase9, as well as decreased the expression of the antiapoptotic protein Bcl2, compared with the control group (*p* < 0.01). In contrast, MRS treatment significantly increased the expression of Bcl2 and decreased the expression of Bax and cleaved caspase9 (*p* < 0.05) (Figures 6(c) and 6(d)). These results revealed that MRS treatment could significantly suppress neuron apoptosis after CA/CPR.

### 3.6. MRS Ameliorated the ER Stress in the Hippocampus after CA/CPR

**Figure 7:**
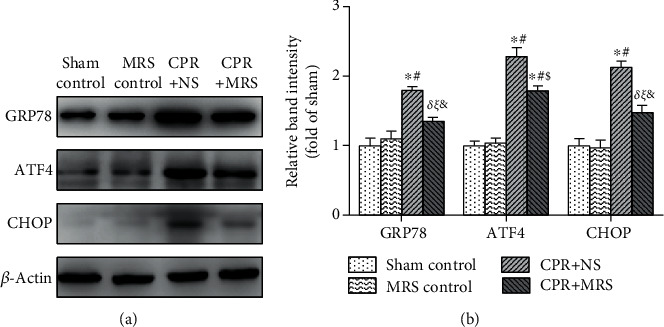
MRS ameliorated the ER stress and related apoptosis process in the hippocampus after CA/CPR. (a) ER stress-associated apoptosis signaling pathway protein GRP78, ATF4, and CHOP expression levels. (b) The relative band intensities (fold of the sham control group). Data were reported as mean ± SD. The statistical significance was assessed by the one-way ANOVA test (^*δ*^*p* < 0.05, and ^∗^*p* < 0.01 vs. sham control group; ^*ξ*^*p* < 0.05 and ^#^*p* < 0.01 vs. MRS control group; ^&^*p* < 0.05 and ^$^*p* < 0.01 vs. CPR+NS group).

The molecular mechanisms of apoptosis were further clarified through the detection of the expression level of GRP78/ATF4/CHOP by western blot (Figure 7(a)). The results showed that compared with the control group, the CPR+NS group had significantly higher expression levels of the ER stress biomarkers GRP78 and ATF-4, as well as the ER-induced apoptosis-related molecule CHOP (*p* < 0.01), whereas MRS treatment reversed these results (*p* < 0.05) (Figure 7(b)).

### 3.7. MRS Attenuated the Levels of Oxidative Stress in the Hippocampus after CA/CPR

**Figure 8:**
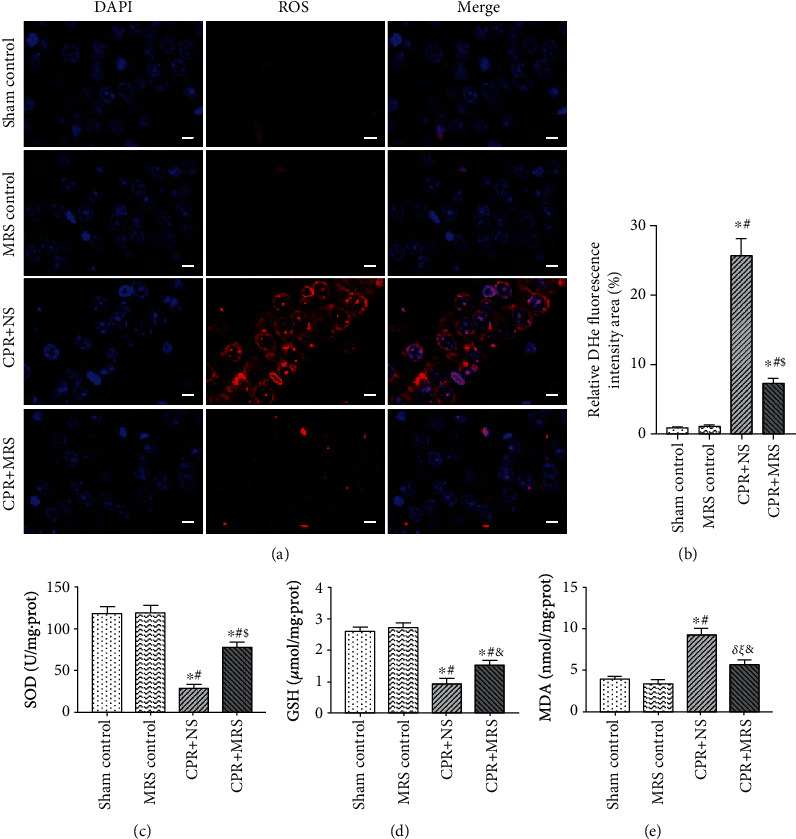
MRS attenuated oxidative stress in the hippocampus after CA/CPR. (a) The DHE fluorescence staining of brain tissue cryosection. (b) The ROS relative fluorescence intensity area (fold of sham control group). The levels of (c) SOD, (d) GSH, and (e) MDA in hippocampal tissue were assessed. Scale bars indicated 25 *μ*m. Data were reported as mean ± SD. The statistical significance was assessed by the one-way ANOVA test (^*δ*^*p* < 0.05, and ^∗^*p* < 0.01 vs. sham control group; ^*ξ*^*p* < 0.05, and ^#^*p* < 0.01 vs. MRS control group; ^&^*p* < 0.05 and ^$^*p* < 0.01 vs. CPR+NS group).

Oxidative stress plays a crucial role in triggering brain injury by CA. Studies have shown that massive ROS were produced during tissue ischemia-reperfusion injury [11]. In addition, studies have shown that excessive ROS triggers ER stress overload and induces CHOP-mediated activation of apoptosis. Meanwhile, ER stress overload also contributes to detrimental oxidative stress. Therefore, we examined the levels of ROS, antioxidant factor SOD, GSH, and oxidant factor MDA in each group of hippocampal tissues. We detected the intracellular ROS levels by DHE fluorescence staining (Figures 8(a) and 8(b)). The results demonstrated that the DHE fluorescence intensity was weak in the control group and significantly increased after CA/CPR challenge (*p* < 0.01), but the intensity of DHE fluorescence was significantly diminished in the CPR+MRS group versus the CPR+NS group (*p* < 0.01). The ELISA results indicated that the levels of SOD and GSH were significantly reduced and MDA levels were obviously higher in the CPR+NS group than those in the control group (*p* < 0.01). However, MRS treatment reversed this change, with elevated SOD and GSH activity and reduced MDA activity in the CPR+MRS group, compared to the CPR+NS group (*p* < 0.05) (Figures 8(c)–8(e)). Based on these results, MRS exerts an antioxidative effect on brain injury after CA/CPR.

## 4. Discussion

Cardiac arrest accounts for fifteen percent of all mortality, and the survival rate for discharge from hospital is less than twenty percent, representing a serious threat for public health [26]. Brain injury after CPR is the primary causality of death and poor prognosis in patients. The pathogenesis of brain injury after CPR is complicated and is primarily related to inflammatory responses, oxidative stress, and apoptosis, leading to severe neuronal damage and neuronal death [2]. While numerous studies have been conducted on brain injury after CPR, these studies mainly focus on the underlying mechanisms. The availability of treatments for brain injury after CA/CPR remains limited [27]. Therefore, it is essential to develop a new therapeutic method to alleviate brain injury in the early period and promote the survival rate of patients with ROSC.

Early fluid rehydration and volume expansion play an essential role in sustaining blood pressure and reducing mortality after ROSC [1]. Methane-rich saline was prepared by dissolving methane in sterile saline at high pressure, which has the same osmolality as saline, enabling it to be used as a therapeutic fluid after CPR. It is well known that methane and hydrogen possess similar biological activities. Hayashida and colleague's study has shown that inhalation of hydrogen after ROSC was effective to attenuate neurological damage in a rat model of CA/CPR [28]. Gao et al. have shown that hydrogen-rich saline significantly improved the survival and neurological function of CA/CPR rats by inhibiting ER stress [29]. Studies have demonstrated that methane exerts a protective role in various disease models related to inflammation, oxidative stress, and apoptosis [30]. However, no studies yet have clarified the role of methane in brain injury after CA/CPR.

In this study, we evaluated the ability of methane as a therapeutic fluid after ROSC and explored its potential mechanism of protection against brain injury after CA/CPR. Asphyxial cardiac arrest is an excellent model for simulating clinical cardiac arrest cases. We successfully constructed an asphyxial CA model by clamped rat endotracheal intubation for 6 min. According to the results presented in this study, MAP was significantly declined after CA/CPR, while MRS treatment helped to stabilize and improve MAP and HR post-ROSC in the early stages. This result was similar to the results of previous studies [31]. The hippocampal CA1 region is most sensitive to cerebral ischemia and hypoxia, allowing the pathological changes to be observed at an early stage. At 6 h post-ROSC, Nissl staining and NeuN immunohistochemical staining revealed that the number of viable neuronal cells in the hippocampus CA1 region was decreased, whereas neuronal death was significantly diminished after MRS treatment. Thus, we would like to consider MRS as an early therapeutic resuscitation fluid that can stabilize vital signs and reduce neuronal damage caused by ischemia and hypoxia after CA/CPR.

The inflammatory response is a key mechanism of ischemia-reperfusion injury [24]. Following ischemic-hypoxic injury of brain tissue, the levels of IL-1*β*, IL-6, TNF-*α*, and other inflammatory factors increase dramatically, which can further induce the expression of adhesion molecules and promote the transendothelial migration of neutrophils and monocytes, causing leukocytes and platelets to accumulate in the capillaries and thus reducing cerebral blood flow. As well, these inflammatory factors can also exfiltrate into the brain parenchyma, releasing neurotoxic substances such as proinflammatory factors, chemokines, and free radicals, which will damage neurons directly. Studies have shown that during reperfusion injury, large amounts of ROS were produced, which triggered microglial activation, and then activated microglia could produce proinflammatory cytokines that further aggravated brain injury by recruiting inflammatory cell aggregates [8]. On the other hand, excessive production of proinflammatory factors would also further lead to the activation of microglia, creating a vicious cycle [10, 25]. Hayashida et al. have shown that inhalation of hydrogen inhibited microglial cell activation in the hippocampus CA1 region [28]. In our study, it was also observed that MRS treatment inhibited microglial cell activation with a concomitant reduction in the expression of inflammatory factors TNF-*α*, IL-6, and iNOS. The results were consistent with previous studies [32]. It suggests that MRS may alleviate brain injury after CA/CPR by inhibiting microglial activation and reducing inflammatory response.

A growing number of studies suggest that apoptosis is an essential player in neurological dysfunction after brain ischemia-reperfusion injury [5, 33, 34]. Therefore, we detected apoptosis by TUNEL staining and found that more TUNEL-positive cells were present after CA/CPR than in the control group, whereas MRS treatment revealed a lower rate of TUNEL-positive cells, which is consistent with previous studies. Western blot detected the expression levels of apoptosis-related proteins such as Bax, Bcl2, and caspase9 in hippocampal tissues that also validated this result. It is widely recognized that the ER plays a crucial role in the maintenance of calcium homeostasis. After ischemia-reperfusion injury, calcium overload and free radical production often trigger ER stress. Dramatic ER stress may deteriorate cell function and convert adaptation programs into CHOP-mediated apoptosis, cleaning out irreversibly damaged cells. Therefore, we further investigated ER stress and induced apoptosis-related proteins. It showed that MRS treatment downregulated the expression of GRP78, ATF4, and CHOP, which were enhanced after CA/CPR. The results were consistent with previous studies [21] and suggested that MRS could reduce neuronal cell death by inhibiting ER stress-regulated apoptosis.

**Figure 9:**
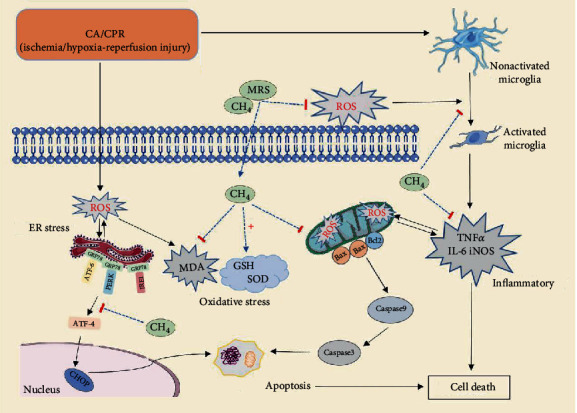
The underlying mechanism for MRS protection against CA/CPR brain injury.

Previous studies have shown that the excessive ROS generated after ischemia-reperfusion injury could activate microglia and trigger ER stress [11, 35]. To clarify the mechanism of microglial activation and ER stress induced by ischemia-reperfusion injury after CA/CPR, we examined ROS levels in hippocampal tissues. Consistent with previous findings, our study demonstrated that MRS treatment dramatically reduced ROS levels of neuron cells in the hippocampal CA1 region. Further, we measured the levels of GSH, SOD, and MDA in hippocampal tissues. The results showed that MRS treatment significantly increased the GSH and SOD levels and decreased the MDA levels. We speculated that MRS could reduce oxidative stress, inhibit microglial activation-induced inflammatory responses, and attenuate ER stress-mediated apoptosis by inhibiting ROS generation. The underlying mechanism for MRS to play a protective role is schematized in Figure 9.

However, the exact molecular mechanisms of methane remain elusive. Several hypotheses have been proposed by Fink to explain the biological effects of methane [36]. Fink speculated that the changes in cellular receptors, specific oxygenases, and cellular redox steady state might be related to the mechanism of the biological effects of methane [36]. Recently, Tuboly et al. have demonstrated that phosphatidylcholine was broken into methane to neutralize ROS after mitochondrial dysfunction, improving the redox imbalance state of the organism [37]. As a consequence, the exogenous administration of methane may exert a series of protective effects by neutralizing the ROS induced by ischemic hypoxia, but further studies would be required.

In conclusion, this study showed that MRS treatment can attenuate post-CA/CPR brain injury by inhibiting oxidative stress, microglial activation-induced inflammatory responses, and ER stress-related apoptosis. These results provide a foundation of methane treatment for early post-CA/CPR brain injury, suggesting that MRS may have promising clinical application as an effective therapeutic resuscitation fluid for early post-ROSC. However, a considerable amount of clinical research remains to be performed before MRS could be launched into clinical practice.

## Figures and Tables

**Table 1:** Baseline characteristics and physiological parameters.

	Sham control	MRS control	CPR+NS	CPR+MRS
Baseline				
Body weight (g)	297.3 ± 17.3	295.5 ± 12.8	301.9 ± 16.5	293.7 ± 15.0
HR (times/min)	400.1 ± 40.4	391.6 ± 35.3	386.3 ± 38.1	393.9 ± 38.1
MAP (mmHg)	100.3 ± 14.3	95.1 ± 10.8	98.8 ± 14.2	95.8 ± 13.5
Cardiac arrest model				
Asphyxia time (s)			79.8 ± 36.9	81.2 ± 30.2
Resuscitation time (s)			404.3 ± 80.3	391.5 ± 67.1
ROSC rate (%)			75.0	73.9
Post-ROSC mortality (%)			33.3	29.4

Data were reported as mean ± SD.

## Data Availability

The data related to rat model data, biochemical index detection, histological staining, and western blot images used to support the findings of this study are available from the corresponding authors upon request.

## Abbreviations

CA: Cardiac arrest
CPR: Cardiopulmonary resuscitation
ROSC: Recovery of spontaneous circulation
MRS: Methane-rich saline
NS: Normal saline
ER: Endoplasmic reticulum
MAP: Mean arterial pressure
NeuN: Hexaribonucleotide-binding protein 3
Iba1: Ionized calcium-binding adapter molecule 1
TMEM119: Transmembrane protein 119
CD68: Cluster of differentiation 68
TNF-*α*: Tumor necrosis factor-alpha
IL-6: Interleukin-6
IL-10: Interleukin-10
iNOS: Inducible nitric oxide synthase
GRP78: Glucose-regulating protein 78
ATF4: Activating transcription factor 4
CHOP: C/EBP homologous protein
ROS: Reactive oxygen species
GSH: Glutathione
SOD: Superoxide dismutase
MDA: Malondialdehyde.
